# Supplementary material for: The interactome of intact mitochondria by cross-linking mass spectrometry provides evidence for coexisting respiratory supercomplexes
Source: Mol Cell Proteomics. 2017 Dec 8;17(2):216–32. doi: 10.1074/mcp.RA117.000470 (PMC5795388; doi:10.1074/mcp.RA117.000470)
Supplement: Supplemental Data [file supp_RA117.000470_133922_0_supp_23978_fzffwf.zip › spectra_annotation/mito_spectra_annotation/390-1-5-1-6-1.pdf]

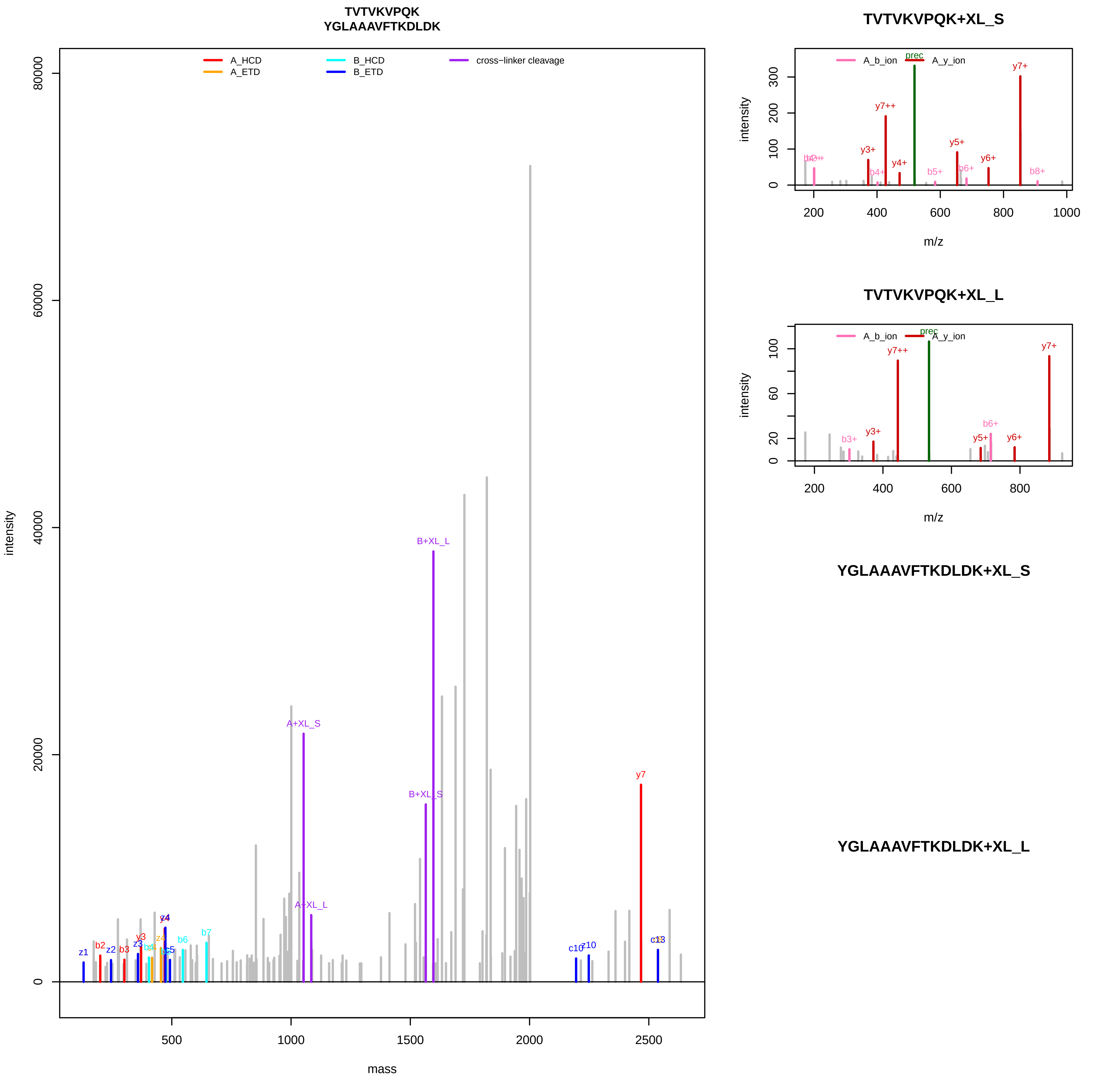
TVTVKVPQK
YGLAAAVFTKDLDK
A_HCD
A_ETD
B_HCD
B_ETD
cross−linker cleavage
intensity
mass
0
20000
40000
60000
80000
500
1000
1500
2000
2500
z1
b2
z2
b3
y3
z3
b4
z4
y4
c5
b6
b7
A+XL_S
A+XL_L
B+XL_L
B+XL_S
c10
z10
y7
c13
TVTVKVPQK+XL_S
A_b_ion
A_y_ion
prec
y7+
y7++
y3+
y4+
b4+
b5+
y5+
b6+
y6+
b8+
m/z
TVTVKVPQK+XL_L
b3+
YGLAAAVFTKDLDK+XL_S
YGLAAAVFTKDLDK+XL_L
